# Chemosensitizing activities of cyclotides from *Clitoria ternatea* in paclitaxel-resistant lung cancer cells

**DOI:** 10.3892/ol.2012.1042

**Published:** 2012-11-22

**Authors:** ZHANG SEN, XIAO KAI ZHAN, JIN JING, ZHANG YI, ZHOU WANQI

**Affiliations:** 1 State Key Laboratory of Bioactive Substance and Function of Natural Medicines, Institute of Materia Medica, Chinese Academy of Medical Sciences and Peking Union Medical College, Beijing 100050;; 2 Department of Hematology and Oncology, Chaoyang Hospital, Capital Medical University, Beijing 100043, P.R. China

**Keywords:** cyclotides, chemosentization, MTT assay, *Clitoria ternatea*, paclitaxel-resistant

## Abstract

Cyclotides comprise a family of circular mini-peptides that have been isolated from various plants and have a wide range of bioactivities. Previous studies have demonstrated that cyclotides have antitumor effects and cause cell death by membrane permeabilization. The present study aimed to evaluate the cytotoxicity and chemosensitizing activities of cyclotides from *Clitoria ternatea* in paclitaxel-resistant lung cancer cells. In this study, a total of seven cyclotides were selected for colorimetric cell viability assay (MTT assay) to evaluate their anticancer and chemosensitizing activities in the lung cancer cell line A549 and its sub-line A549/paclitaxel. Results suggested that certain cyclotides had significant anticancer and chemosensitizing abilities; such cyclotides were capable of causing multi-fold decreases in the half maximal inhibitory concentration (IC_50_) value of cliotides in the presence of paclitaxel. More importantly, their bioactivities were found to be correlated with their net charge status. In conclusion, cyclotides from *C. ternatea* have potential in chemosensitization application.

## Introduction

An abundance of cyclotides have been isolated and confirmed from *Clitoria ternatea* (Fabaceae family) (1,2). The concentration of cyclotides from this plant is relatively higher than that of the majority of cyclotide-containing plants from the Rubiaceae and Violacecae families. *C. ternatea* is therefore an ideal resource for investigating the bioactivities of cyclotides. In previous studies, *in vitro* cell-based assays have demonstrated that certain cyclotides have significant cytotoxic activities by disrupting the cell membrane integrity (3,4). By contrast, in an *in vivo* study, even the most potent cyclotide, cycloviolacin O2, did not demonstrate a significant anticancer effect in a xenograft model (5). Gerlach *et al* revealed that cyclotides may have potential chemosensitizing abilities when combined with other anticancer reagents (6). Cycloviolacin O2 and psyle cyclotides from *Psychotria leptothyrsa* were used to treat the doxorubicin-resistant human breast cancer cell line MCF-7/ADR. It was found that cyclotides were capable of enhancing the doxorubicin effect that inhibits MCF-7/ADR proliferation; coexposure caused the half maximal inhibitory concentration (IC_50_) value to decrease 4- to 7-fold compared with single doxorubicin. The mechanism was considered to be associated with a leakage of cell membrane caused by cyclotides, thus enabling the chemical reagent to pass through the cell membrane. Therefore, we hypothesize that this type of chemosensitizating ability may be wide spectrum with less selectivity. In the present study, the human lung cancer cell line A549 and its sub-line A549/paclitaxel were selected to provide support for this hypothesis. The role of the net charge status of cyclotides on cytotoxicity and chemosentization was also investigated.

## Materials and methods

### Isolation and purification of cyclotides

Cyclotides from *C. ternatea*, termed specifically as cliotides, were isolated and purified from *C. ternatea* as described previously (2). Briefly, flowers and seeds were ground, and then extracted by 20% ethanol (V/V, with water). Crude extract was centrifuged for 30 min at 10,000 x g and the supernatant was passed through a 0.45 *μ*m filter to clear the debris. Different types of cliotide peptides with different acetonitrile concentration gradients were fractionated and purified by RP-HPLC. Based on the amino acid sequence and net charges, a total of seven cliotide peptide molecules were selected for use in the present study (Table I).

### Cell culture

Human non-small lung cancer (A549) cells were obtained from the American Type Culture Collection (ATCC; Manassas, VA, USA). An A549-derived paclitaxel-resistant sub-line, A549/taxol, was established by the Institute of Materia Medica, Chinese Academy of Medical Sciences (Beijing, China). For maintenance, cells were cultured in RPMI-1640 medium (Gibco Laboratories, Life Technologies Inc.; Grand Island, NY, USA) with 10% fetal bovine serum (FBS; Gibco) at 37°C in a humidified 5% CO_2_ atmosphere.

### Cytotoxicity assay

Various types of cliotides were assessed for their cytotoxicity activity in A549 cells and their chemosensitizating capability in A549/taxol cells, as previously described by Gerlach *et al*(6). Briefly, cells were seeded in 96-well flat-bottomed microtiter plates (1×10^4^). After culture for 4 h, cells were treated with cliotide T2 (CT2), CT4, CT7, CT10, CT12, CT19, CT20 and paclitaxel at 0.4, 1, 2, 4, 10 and 20 *μ*M, in 100 *μ*l of media for 72 h. The medium was removed from each well and 100 *μ*l of 3-(4,5-dimethylthiazol-2-yl)-2,5-diphenyl tetrazolium bromide (MTT; 0.5 mg/ml in PBS) was added in the absence of light; formazan crystals were produced over a 4 h incubation period. To dissolve crystals, 150 *μ*l of 0.04 N HCl in isopropanol was added to each well and the optical density at 540 nm was measured on a Tecan Spectrophotometer (Tecan SPECTRAFluor, Tecan, Männedorf, Switzerland). For coexposure experiments, the same protocol was followed but concentrations were modified to 50:50 ratios of each cliotide and paclitaxel at the following concentrations: 0.2, 0.5, 1, 2, 5 and 10 *μ*M. Each test was performed with 3 replicates and repeated in triplicate. Replicates were averaged and the blank subtracted from control and test concentrations. Optical density of the treated wells was compared with that of the controls (100% survival); percentage cell survival was calculated and plotted by GraphPad Prism (GraphPad Software, Inc.; San Diego, CA, USA).

### Statistical analysis

All data were analyzed with GraphPad Prism 4.0 software. The IC_50_ values (*μ*M) of each treatment were calculated and a combination of paired t-tests and analysis of variance (ANOVA) were performed. P<0.05 was considered to indicate a statistically significant difference.

## Results

### Cytotoxicity of cliotides

**Figure 1.**
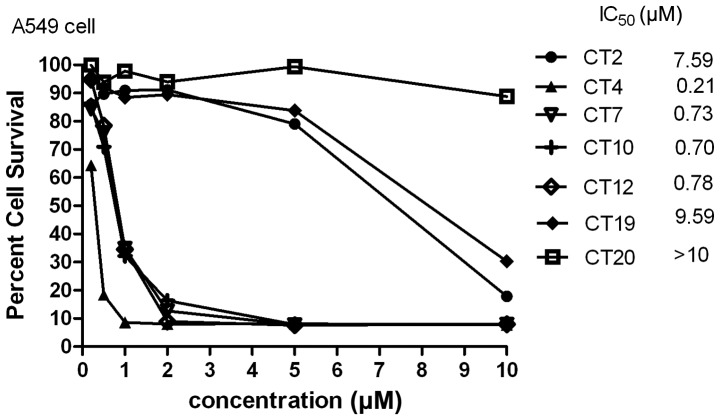
Survival index curves of CT2, CT4, CT7, CT10, CT12, CT19 and CT20 at six different concentrations in the human cancer cell line A549. Each point represents the mean ± standard error, calculated using nonlinear regression in GraphPad Prism.

All cliotides in Table I were used to test cytotoxicity in A549 cells. All of these demonstrated significant cytotoxicity, with the exception of CT19 and CT20 whose predicted IC_50_ values were ∼10 *μ*M (Fig. 1). Previous studies have suggested that net charge is an important factor in cytotoxic activity, and bracelet cyclotides were generally more cytotoxic than Möbius cyclotides (7). In the present study, CT4, which had a net charge of +2, had the lowest IC_50_ value (0.21 *μ*M) compared with the other cyclotides. However, a positive charge of >2 did not increase the cytotoxicity; CT19 had a charge of +3 and its IC_50_ value was ∼10 *μ*M, while CT20 had a charge of +4, with an IC_50_ value above 10 *μ*M.

### Chemosensitization of cliotides with paclitaxel on A549/paclitaxel

Based on the results above, cliotides which had significant cytotoxicity in A549 cells (IC_50_<10 *μ*M) were selected for coexposure experiments in the A549 paclitaxel-resistant cell line. These cliotides were CT2, CT4, CT7, CT10 and CT12.

**Figure 2.**
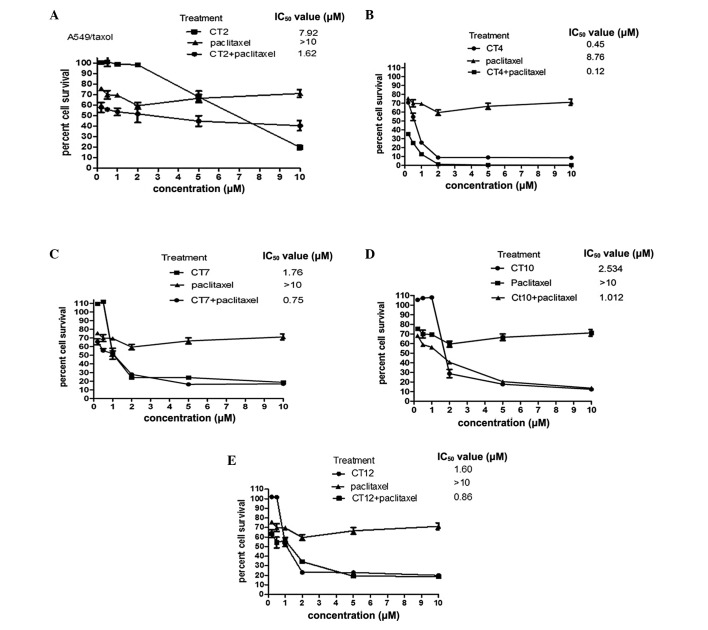
Chemosensitizing effect of cliotides on paclitaxel resistant A549 cells. The data are representative of three independent experiments. All cliotides demonstrated a significant chemosensitizing effect. Analysis of variance for each graph: P<0.05.

Fig. 2A–E and Table II summarize the chemosensitizing effect of five types of cliotide, all of which demonstrated positive results reflected by the multi-fold decreases in the IC_50_ value of cliotides in the presence of paclitaxel. A four-fold decrease in the CT2-treated group, a three-fold decrease in the CT4-, CT7- and CT10-treated groups and a one-fold decrease in the CT12-treated group were observed.

## Discussion

At present, >200 cyclotides have been isolated and identified from various plants of the Violaceae, Rubiaceae, Cucurbitaceae and Fabaceae families (8–11). Cyclotides comprise a family of circular mini-proteins and have a wide range of bioactivities. They have a characteristic head-to-tail cyclized backbone typically composed of 28–37 amino acids, and a knotted disulfide topology involving six conserved cysteine residues (12–14). The cyclic backbone and conserved cysteine residues form a unique structure termed the cyclic cystine knot (CCK), in which two of the disulfide bonds and their connecting backbone segments form an embedded ring that is penetrated by the third disulfide bond (14). Aside from being a characteristic structural feature of the cyclotide family, the CCK motif enables these mini-proteins to be exceptionally resistant to chemical, enzymatic and thermal degradation (15). Therefore, cyclotides have become an ideal scaffold for drug delivery (15,16). Cyclotides exhibit a range of biological activities, including uterotonic (17), hemolytic (18), antineurotensin (19), anti-HIV (20), cytotoxic (3) and antibacterial (18) activities; however, their natural function is considered to be as plant defence molecules, based on their insecticidal (21) and molluscidal (22) properties.

Among all the bioactivities of cyclotides, cytotoxicity was was one of the first to be identified and has been investigated widely. However, the sharp dose-response profile and poor *in vivo* anticancer results affected the usage of cyclotides in clinical treatment (5). In the present study, consistent with previous research, cliotides were less cytotoxic against A549/paclitaxel compared to A549 (Table II). Additionally concordant, cliotides with a charge of +2 were the most cytotoxic; CT4 had the lowest IC_50_ value. The cytotoxic ranking was IC_50_ CT4 (+2)<IC50 CT7 (+1) and CT12 (+1)<IC_50_ CT10 (0)<IC_50_ CT2 (−1); cytotoxicity increased with an increase in net positive charge. However, CT19 and CT20, which had charges of +3 and 4, respectively, did not show significant cytotoxicity. With regard to why higher cliotide positive charges may decrease cytotoxicity, we hypothesized that although multiple positive charges made it easier for the cliotides to adhere to the cell membrane, they also changed the peptide space structure and reduced the hydrophobicity, thus reducing the ability to disrupt the cell membrane.

Drug resistance is one of the greatest obstacles limiting chemotherapy in clinical treatment. The chemosensitizing ability of the cliotides in the A549/paclitaxel cells in the present study is notable; all five tested cyclotides demonstrated a two- to four-fold decrease in IC_50_ value in the presence of paclitaxel.

In summary, this study demonstrated that cyclotides from *C. ternatea* have the potential to be used in chemosensitization for treating cancer. We are optimistic that cyclotides are suitable drug candidates after modification and their charge status plays a significant role in their bioactivities.

## Figures and Tables

**Table I.** Cliotides involved in the present study.

Cliotide	Sequence	MW^a^	Net charge	Subfamily
CT2	GEFLK**C**GES**C**VQGE**C**–YT– –PG**C**S**C**DWPI**C**KKN	3260	−1	M
CT4	GIP– – **C**GES**C**VFIP**C**–ITAAIG**C**S**C**KSKV**C**YRN	3098	+2	B
CT7	GIP– – **C**GES**C**VFIP**C**TVTALLG**C**S**C**KDKV**C**YKN	3227	+1	B
CT10	GVP– –**C**AES**C**VWIP**C**TVTALLG**C**S**C**KDKV**C**YLN	3251	0	B
CT12	GIP– –**C**GES**C**VYIP**C**TVTALLG**C**S**C**KDKV**C**YKN	3243	+1	B
CT19	GSVIK**C**GES**C**LLGK**C**–YT– –PG**C**T**C**SRPI**C**KKD	3125	+4	B
CT20	GSAIR**C**GES**C**LLGK**C**–YT– –PG**C**T**C**DRPI**C**KKN	3152	+3	B

a MW, molecular weight, reported as monoisotopic mass; B, bracelet; M, Möbius. Cysteine residues are in bold.

**Table II.** Half maximal inhibitory concentration (IC_50_*μ*M) of cliotides in the human lung cell line (A549) and its drug resistant cell (A549/paclitaxel).

Cyclotides	A549	A549/paclitaxel	Coexposure to paclitaxel
CT2	7.59	7.92	1.62
CT4	0.21	0.45	0.12
CT7	0.73	1.76	0.75
CT10	0.70	2.53	1.01
CT12	0.78	1.6	0.86
Paclitaxel	1.21	>10	>10

## References

[1] Poth AG, Colgrave ML, Philip R (2011). Discovery of cyclotides in the fabaceae plant family provides new insights into the cyclization, evolution, and distribution of circular proteins. ACS Chem Biol.

[2] Nguyen GK, Zhang S, Nguyen NT (2011). Discovery and characterization of novel cyclotides originated from chimeric precursors consisting of albumin-1 chain a and cyclotide domains in the Fabaceae family. J Biol Chem.

[3] Lindholm P, Göransson U, Johansson S (2002). Cyclotides: a novel type of cytotoxic agents. Mol Cancer Ther.

[4] Herrmann A, Svangård E, Claeson P, Gullbo J, Bohlin L, Göransson U (2006). Key role of glutamic acid for the cytotoxic activity of the cyclotide cycloviolacin O2. Cell Mol Life Sci.

[5] Burman R, Svedlund E, Felth J (2010). Evaluation of toxicity and antitumor activity of cycloviolacin O2 in mice. Biopolymers.

[6] Gerlach SL, Rathinakumar R, Chakravarty G (2010). Anticancer and chemosensitizing abilities of cycloviolacin O2 from *Viola odorata* and psyle cyclotides from *Psychotria leptothyrsa*. Biopolymers.

[7] Svangård E, Göransson U, Hocaoglu Z, Gullbo J, Larsson R, Claeson P, Bohlin L (2004). Cytotoxic cyclotides from *Viola tricolor*. J Nat Prod.

[8] He W, Chan LY, Zeng G, Daly NL, Craik DJ, Tan N (2011). Isolation and characterization of cytotoxic cyclotides from *Viola philippica*. Peptides.

[9] Kaas Q, Craik DJ (2010). Analysis and classification of circular proteins in CyBase. Biopolymers.

[10] Mulvenna JP, Wang C, Craik DJ (2006). CyBase: a database of cyclic protein sequence and structure. Nucleic Acids Res.

[11] Wang CK, Kaas Q, Chiche L, Craik DJ (2008). CyBase: a database of cyclic protein sequences and structures, with applications in protein discovery and engineering. Nucleic Acids Res.

[12] Yeshak MY, Burman R, Asres K, Göransson U (2011). Cyclotides from an extreme habitat: characterization of cyclic peptides from *Viola abyssinica* of the Ethiopian highlands. J Nat Prod.

[13] Craik DJ (1999). Applications of NMR in drug design: Structure-activity relationships in disulfide-rich peptides. Protein Peptide Lett.

[14] Göransson U, Craik DJ (2003). Disulfide mapping of the cyclotide kalata B1. Chemical proof of the cystic cystine knot motif. J Biol Chem.

[15] Colgrave ML, Craik DJ (2004). Thermal, chemical, and enzymatic stability of the cyclotide kalata B1: The importance of the cyclic cystine knot. Biochemistry.

[16] Wong CT, Taichi M, Nishio H, Nishiuchi Y, Tam JP (2011). Optimal oxidative folding of the novel antimicrobial cyclotide from *Hedyotis biflora* requires high alcohol concentrations. Biochemistry.

[17] Gran L, Sandberg F, Sletten K (2000). *Oldenlandia affinis* (R&S) DC. A plant containing uteroactive peptides used in African traditional medicine. J Ethnopharmacol.

[18] Tam JP, Lu YA, Yang JL, Chiu KW (1999). An unusual structural motif of antimicrobial peptides containing end-to-end macrocycle and cystine-knot disulfides. Proc Natl Acad Sci USA.

[19] Witherup KM, Bogusky MJ, Anderson PS (1994). Cyclopsychotride A, a biologically active, 31-residue cyclic peptide isolated from *Psychotria longipes*. J Nat Prod.

[20] Gustafson KR, McKee TC, Bokesch HR (2004). Anti-HIV cyclotides. Curr Protein Pept Sci.

[21] Barbeta BL, Marshall AT, Gillon AD, Craik DJ, Anderson MA (2008). Plant cyclotides disrupt epithelial cells in the midgut of lepidopteran larvae. Proc Natl Acad Sci USA.

[22] Plan MR, Saska I, Cagauan AG, Craik DJ (2008). Backbone cyclised peptides from plants show molluscicidal activity against the rice pest *Pomacea canaliculata* (golden apple snail). J Agric Food Chem.

